# Considerable MHC Diversity Suggests That the Functional Extinction of Baiji Is Not Related to Population Genetic Collapse

**DOI:** 10.1371/journal.pone.0030423

**Published:** 2012-01-17

**Authors:** Shixia Xu, Jianfeng Ju, Xuming Zhou, Lian Wang, Kaiya Zhou, Guang Yang

**Affiliations:** Jiangsu Key Laboratory for Biodiversity and Biotechnology, College of Life Sciences, Nanjing Normal University, Nanjing, China; Australian Wildlife Conservancy, Australia

## Abstract

To further extend our understanding of the mechanism causing the current nearly extinct status of the baiji (*Lipotes vexillifer*), one of the most critically endangered species in the world, genetic diversity at the major histocompatibility complex (MHC) class II *DRB* locus was investigated in the baiji. Nine highly divergent *DRB* alleles were identified in 17 samples, with an average of 28.4 (13.2%) nucleotide difference and 16.7 (23.5%) amino acid difference between alleles. The unexpectedly high levels of *DRB* allelic diversity in the baiji may partly be attributable to its evolutionary adaptations to the freshwater environment which is regarded to have a higher parasite diversity compared to the marine environment. In addition, balancing selection was found to be the main mechanisms in generating sequence diversity at baiji *DRB* gene. Considerable sequence variation at the adaptive MHC genes despite of significant loss of neutral genetic variation in baiji genome might suggest that intense selection has overpowered random genetic drift as the main evolutionary forces, which further suggested that the critically endangered or nearly extinct status of the baiji is not an outcome of genetic collapse.

## Introduction

**Figure 1:**
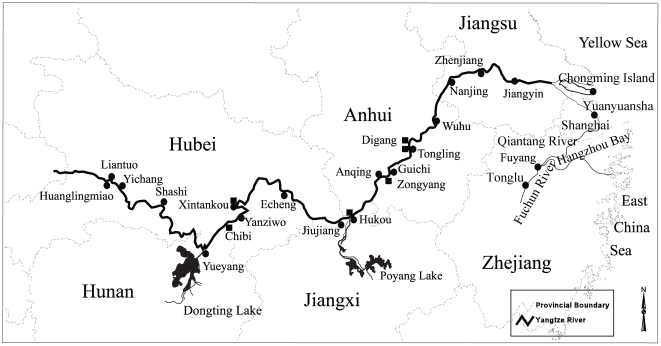
Historical distribution of the baiji in main Yangtze River and neighbouring water bodies. The dot indicates baiji distribution by 1980s inferred from survey data (1979–1981) by Zhou [7]; the square indicates the remnant habitatof the baiji inferred from 1997 survey data by Zhang et al. [8].

The baiji or Yangtze River dolphin (*Lipotes vexillifer*), endemic to the Yangtze River of China, has long been recognized as one of the most endangered species in the world [1]–[4]. This species historically occurred along approximately 1700 km of the middle and lower reaches of the Yangtze River from Yichang to the estuary near Shanghai, and also in two large appended lakes (Dongting Lake and Poyang Lake), and the neighbouring River such as Qiantang River and Fuchun River (Figure 1) [5]–[7]. However, it has dramatically declined in the past decades and is now regarded as the first large mammal species to have become extinct in over 50 years [3]. The first report regarding population abundance based on quantitative survey data (1979–81) was made by Zhou (Figure 1) [7], which estimated about 400 animals all told. However, attempted comprehensive surveys of the entire species' range in 1997–99 resulted in a maximal count (November 1997) of 13 dolphins (Figure 1) [8], leading to the generally accepted view that abundance had continued to decline and that the total population was by that time very small. Although great efforts have been made in the past decades to conserve this critically endangered species, a recent field expedition conducted in 2006 failed to find any baiji individual in the wild, strongly suggesting that this species is now likely to be extinct [3]. It is possible that one or two animals might have been missed, but we have to accept the fact that the baiji is functionally extinct. The primary factors driving its decline include entanglement in fishing gears, electrocution from electric fishing, collisions with vessels, and blasting for channel maintenance or harbour construction [1], [9]–[10]. Specially, the baiji's range is believed to have progressively diminished through loss of peripheral subpopulations followed by large-scale range contraction and fragmentation (Figure 1) [1], [11]. In addition, emerging infectious diseases, particularly parasites infection aggravated by pollution, is probably another major factor contributing to population decline.

Cetaceans are warm-blooded and breathe air like other mammals, however, they rely on the aquatic environment for their life needs. This may result in an interaction with both terrestrial and marine pathogen risks [12]. In the last 20 years, thousands of marine mammals have died due to epizootics caused by viral infections [13]. It is well known that baiji has a very limited distribution endemic to the Yangtze River, the freshwater environment might have a relatively higher level of pathogens than marine waters, although systematic environmental studies have yet to be conducted. Furthermore, incidentally captured/killed individuals had helminth infestations in the stomach [7], which suggested that parasite infections might be another factor for the population decline.

The major histocompatibility complex (MHC) plays a central role in the development of the immune response against pathogens. MHC class I and class II molecules present peptide epitopes to T-cell receptors initiating the adaptive immune response [14]. In general, large and/or outbred populations typically show abundant polymorphism characterized by divergent alleles at the second exon of MHC class I and class II genes, which is regarded to be maintained by some form of balancing selection [15]–[16]. However, in small populations or endangered species, demographic effects such as population bottleneck and genetic drift usually resulted in limited MHC diversity and increasing species vulnerability of pathpgens and parasites [17]–[18]. This was evidenced in the Spanish ibex *Capra pyrenaica* [19], and Gulf of California endemic porpoise *Phocoena sinus* [20], although a link between the level of MHC variation and the condition of populations or species has not been convincingly demonstrated. On the other hand, other species such as island fox (*Urocyon littoralis*) [21] have maintained moderate to high MHC variability despite severe population bottleneck. Thus, a species' demographic history and parasite-driven balancing selection may therefore play major roles in shaping the pattern of MHC variation [15]–[16], [22]–[23]. In addition, intragenic recombination (or homologous gene conversion) has also been suggested as another important evolutionary mechanism for the generation of MHC sequence diversity [24]. High recombination rate in the MHC enables the host to keep up with the usually faster evolving parasites and might have beneficial fitness effects for the host.

To determine whether MHC variation has been maintained by natural selection and recombination despite the intense genetic drift implied by the low genetic polymorphism at neutral genetic markers, in this study, highly polymorphic exon 2 of MHC class II *DRB* gene was investigated in the baiji. Additionally, we have compared the allelic diversity described in the baiji with those found in other endangered species. Findings from this study will provide basic information for further understanding of how demographic and environmental factors influence MHC variation in the wild animal populations, especially in the endangered species.

## Results

### Allelic diversity

A total of 233 cloned sequences of 215 bp in length were obtained from the sampled 17 individuals. Nine *DRB* unique sequences were identified according to the criteria summarized by Kennedy et al. [25]. None of the sequences contained insertions/deletions (or indels), or stop codons, suggesting that all sequences might come from functional molecules in the genome. No more than two *DRB* unique sequences were detected in each individual, indicating that a single-copy *DRB* locus was amplified from the baiji using this primer sets. These sequences were labeled Live-DRB*01-*09* (Accession number: JN202590-JN202598) according to the nomenclature of Klein et al. [26]. The alleles Live-DRB*01, *02*, *03*, and *04* were common in the baiji and had high frequency from 11.8% to 29.4%, whereas the frequency of rarer alleles ranged from 2.9% to 8.8% (Table 1). The observed heterozygosity (0.71) was less than the expected heterozygosity (0.83) under Hardy-Weinberg proportions (p<0.01, Fisher's exact test), according to a probability test with the Markov chain method. Consequently, significant deviation from HWE was found (*P* = 0.007, SE = 0.0001).

**Table 1:** Alignment of predicted amino acid sequences of baiji MHC *DRB* exon 2 and their allelic frequencies.

	+ + + ++ + + ++ + + ++ + + ++ ++	Allelefrequency
	1112222222 2223333333 3334444444 4445555555 5556666666 6667777777 7777888888 8	
	7890123456 7890123456 7890123456 7890123456 7890123456 7890123456 7890123456 7	
Live-DRB*01	FSNGTERVRF VERHIYNREE YVRFDSDVGE YRAVTELGRR TAEYWNSQKD ILEQNQAALD TYCRHNYEAV E	0.294
Live-DRB*02	....M.Q… LA.YT..AQ. D.H....... F........P D…G.Q… FM..MR.KV. .V..S..WGI G	0.177
Live-DRB*03	.......... .V.RT..... FL........ .....G.... …N...... L…E..KV. .......RV. .	0.118
Live-DRB*04	.........L LVTN…G.. …Y...... H......... .......... L..RRR.EV. .V.....GVG .	0.147
Live-DRB*05	.......... .......... .......... .......... .......... .......... .......... .	0.059
Live-DRB*06	.........L LVTN…G.. …Y...... H........P D......... L..RRR.EV. .V.....GVG .	0.059
Live-DRB*07	.......... .......... FLL....... ....AD.... .......... L..RRR.... .......GVG .	0.088
Live-DRB*08	.........L LVTS…G.. …Y...... H......... .......... L..RRR.EV. .V.....GVG .	0.029
Live-DRB*09	.......... .V.RT..... FL........ .......... …N...... L…E..KV. .......RV. .	0.029

Dots indicate residues identical to the reference sequence of Live-DRB*01. Putative peptide binding sites according to the three dimensional structure of human HLA *DRB* molecules [27] are marked with ‘+’.

A total of 72 of 215 (33.49%) nucleotides and 35 of 71 (49.30%) amino acids were variable (Table 1). The baiji amino acid sequences correspond to sites 16–87 of the human leukocyte antigen (HLA) class II DRB1 sequences [27]. The number of pairwise nucleotide differences between alleles ranged from one (Live-DRB*01 vs. Live-DRB*05) to 57 (Live-DRB*02 vs. Live-DRB*07), whereas amino acid replacements between alleles ranged from 0 (Live-DRB*01 vs. Live-DRB*05) to 30 (Live-DRB*02 vs. Live-DRB*07). Of the 19 PBR (peptide-binding region) sites defined by homology with HLA [27], 11 were variable (57.89%). Twenty-four of 52 (46.15%) sites in the non-PBR were polymorphic, which were mostly located next to the PBR.

### Detecting selection and recombination

Clear signals of positive selection for amino acid replacements in the codons involved in antigen binding were detected at the *DRB* locus. First, the rate of nonsynonymous substitutions was significantly higher than the rate of synonymous substitutions when all sites were included in the analysis (*Z*-test of positive selection, *P* = 0.032) and when only PBR sites were included (*Z*-test, *P* = 0.009; Table 2). Second, the model of codon evolution assuming that a fraction of codons has been affected by positive selection, i.e. M2a, M8 and M3, fit the data substantially better than the uniform ω and nearly neutral models, i.e. M1a, M0 and M7 on the basis of the ΔAIC values (Table 3). The Bayes empirical Bayes (BEB) procedure of model M2a identified only one codons (71-N) as subject to significant positive selection (confidence interval level >0.95), while five codons were subject to significant positive selection under the model M8 with confidence interval level >0.8 (30H, 57T, 71N, 85A, 86Y). These codons are thought to be in the PBR [27].

**Table 2:** The estimated rates of nonsynonymous (*d*_N_) and synonymous (*d*_S_) substitutions for the peptide binding region (PBR) and other regions (non-PBR) and their ratio for *DRB* exon 2 sequences in the baiji.

Positions	Codons	*d*_N_	*d*_S_	*d*_N_ */d*_S_	*P*
PBR	19	0.196(0.059)	0.054(0.028)	3.630	0.009
Non-PBR	52	0.147(0.032)	0.113(0.028)	1.301	0.193
All	71	0.160(0.029)	0.097(0.023)	1.649	0.032

*d*_N_ and *d*_S_ were computed according to the Nei-Gojobori method [55], with standard errors obtained through 1,000 bootstrap replicates in parentheses, and P was the probability that *d*_N_ and *d*_S_ are different using a Z-test.

**Table 3:** Results of maximum-likelihood models for exon 2 of the *DRB* gene in the baiji.

Model code	P	Log-likelihood	ΔAIC	Parameter estimates	Positively selected sites
M0 (one ratio)	1	−684.029	22.788	ω = 0.442, K = 2.268	None
M1a (nearly neutral)	1	−663.348	2.107	p_0_ = 0.455, p_1_ = 0.545, K = 2.704, ω_0_ = 0, ω_1_ = 1.0	Not allowed
M2a (positive selection)	3	−661.388	0.147	p_0_ = 0.450, p_1_ = 0.534, p_2_ = 0.016, K = 2.715, ω_0_ = 0, ω_1_ = 1, ω_2_ = 11.708	71N (0.958)
M3 (discrete)	5	−661.241	Best	p_0_ = 0.455, p_1_ = 0.530, p_2_ = 0.016, K = 2.748, ω_0_ = 0, ω_1_ = 1.188, ω_2_ = 13.405	Not allowed
M7 (beta)	2	−663.560	2.319	p = 0.017, q = 0.016, K = 2.698	Not allowed
M8 (beta and omega)	4	−663.062	1.821	p_0_ = 0.461, p_1_ = 0.539, p_2_ = 0.005, q = 3.074, ω = 1.263, K = 2.753	30H(0.813), 57T(0.832),71N(0.980), 85A(0.822), 86Y(0.836)

Analyses were completed using CodeML (included in the PAML 4 program suite). An alignment of 9 *DRB* sequences (213 bp) from the baiji was used as the input for CodeML. P is the number of parameters in the ω distribution, ω is the selection parameter (*d*_N_/*d*_S_), K estimated transition/transversion rate ratio, and pn proportion of sites that fall into the ωn site class. For models M7 and M8, p and q are the shape parameters of the β function. Positively selected sites were identified in models M2a and M8 by the Bayes empirical Bayes procedure [57]. Sites inferred to be under positive selection are given at the 80% confidence interval level and the levels are given in parentheses. See “Data analysis” and Yang et al. [58] for detailed method description.

The RDP analysis revealed that intragenic recombination was detected in four *DRB* alleles (i.e. Live-DRB*01, Live-DRB*03, Live-DRB*05, and Live-DRB*09) of the 9 *Live-DRB* alleles (Table 4). The parameters of RDP3 were conservatively set to avoid potential false-positive identification of recombination events, so this number represented a minimum of recombinant alleles. Only one potential recombination event was significantly identified (Table 4) using MAXCHI and SISCAN methods, which generated the above four recombinant alleles with Live-DRB*08 as the major parent and Live-DRB*02 as the minor parent. Apparently, some sequence blocks (for example, DNA block 83–198 and DNA block 146–189, see Table 4) were repeatedly involved in recombination events and may have served as recombination hot spots.

**Table 4:** Recombinant sequences at the baiji *DRB* exon 2.

Recombinant sequence	Breakpoint positions			Potential Parent Sequence	Method(Average P-value)	
	Begin	End	Length		MAXCHI	SISCAN
Live-DRB*01	83	198	115	Live-DRB*08/Live-DRB*02 (Unknown)	6.172*10^−1^	8.928*10^−3^
Live-DRB*05	146	189	43	Live-DRB*08/Live-DRB*02 (Unknown)	6.172*10^−1^	8.928*10^−3^
Live-DRB*03	83	198	115	Live-DRB*08/Live-DRB*02 (Unknown)	6.172*10^−1^	8.928*10^−3^
Live-DRB*09	83	198	115	Live-DRB*08/Live-DRB*02 (Unknown)	6.172*10^−1^	8.928*10^−3^

Minor parental sequence: Parent contributing the smaller fraction of sequence, Major parental sequence: Parent contributing the larger fraction of sequence. Unknown: means uncertain parent sequences.

### Phylogenetic analysis

**Figure 2:**
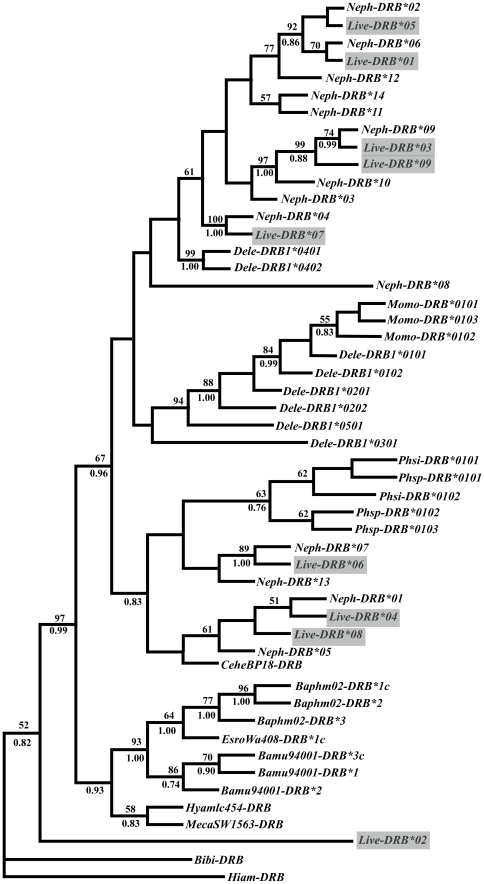
Phylogenetic relationships of the baiji MHC II *DRB* alleles (grey frame) with a representative set of other cetacean alleles. Bootstrap values above 50% (from the neighbour-joining analysis) are shown above respective branches, whereas Bayesian posterior probabilities above 70% are shown below branches. Trees were rooted using *Bison bison*, *Bibi-DRB* (DQ354665) and *Hippopotamus amphibious*, *Hiam-DRB* (EF017820) sequences. Other allelic sequences downloaded from GenBank were included in the analyses, they are: *Balaenoptera musculus* (Bamu94001-DRB*3c: DQ354666; Bamu94003-DRB*1-2: DQ354667-DQ354668); *Balaena mysticetus* (Bamy92005-DRB*1c: DQ354669; Bamy92005-DRB*2c: DQ354671); *B. physalus* (BaphM02-DRB*1c: DQ354672; BaphM02-DRB*2: DQ354673; BaphM08-DRB*3: DQ354674); *C. hectori* (*CeheBP18-DRB*: DQ354675); *Eubalaena australis* (EuauWA9511-DRB2*1c: DQ354676); *Eschrichtius robustus* (EsroWa408-DRB*1c: DQ354678); *Hyperoodon ampullatus* (*HyamIC454-DRB*: DQ354679); *Mesoplodon carlhubbsi* (*MecaSW1563-DRB*: DQ354680); *Delphinapterus leucas* (Dele-DRB1*0101-0501: AF012930-AF012937); *Monodon monoceros* (Momo-DRB*0101-0103: AF012939-AF012941); *P. sinus* (Phsi-DRB*0101-0102: DQ914413-DQ914414); *P. spinipinnis* (Phsp-DRB*0101-0103: DQ914415-DQ914417); *N. phocaenoides* (Neph-DRB*01-14: FJ416157–FJ416170).

Using the AIC criterion, HKY+G was identified as the most appropriate model for the present *DRB* dataset. According to this model, nucleotide frequencies were as follows: 0.225(A), 0.251 (C), 0.339 (G), 0.184 (T), and the gamma shape parameter α = 0.443. The Bayesian tree and NJ tree were separately reconstructed according to this model (HKY+G). Phylogenetic reconstructions of the baiji and other cetacean *DRB* sequences showed moderate bootstrap support for a cetacean-specific clade, distinct from the artiodactyl outgroup sequences such as *Bibi-DRB* and *Hiam-DRB* sequences (Figure 2). Among the cetaceans, there was strong bootstrap support (99%) for the baleen whale clade and odontocete clade but exclusive of Live-DRB*02. Among the odontocete whales, *Live-DRB* alleles did not form a species-specific clade but intermixed with sequences from other speceis. For example, some baiji *DRB* alleles were clustered with alleles of finless porpoise (*Neophocaena phocaenoides*).

## Discussion

### Unexpectedly high levels of *DRB* allelic diversity in the baiji

The baiji is now functionally extinct, which would be the first recorded extinction of a well-studied cetacean species to be directly attributable to human influence [3]. This is a relict species and the only living representative of the family Lipotidae [28]. Its extinction, therefore, would mean not only the loss of a species but of an entire family of river dolphins. In general, low genetic variation, or even lack of, is a common characteristic of many long-term small isolated populations, and is the result of standard evolutionary processes (i.e. genetic drift). This was evidenced in some endangered species, such as vaquita [20]. However, it was surprisingly found in the present study that, contrary to the previous studies with neutral markers, the baiji showed considerable genetic variation at an adaptive MHC locus. A total of nine *DRB* alleles were identified in 17 baiji samples, which is much greater than those of some other marine mammal species/populations examined up to date, e.g. only three *Mian-DRB* alleles in 110 Northern elephant seals (*Mirounga angustrirostris*) [29], eight *Dele-DRB* alleles in 313 beluga [30], and two *Phsi-DRB* alleles in 29 Gulf of California harbour porpoises [20]. Further, the baiji showed a significant *DRB* allelic diversity with an average of 13.2% nucleotide (range 1–57) and 23.5% amino acid (range 0–30) differences between sequences, both of which were comparable to or higher than those found in some other endangered species (Table 5). For example, the average difference in amino acid sequence is 19.2% for the Arabian oryx (*Oryx leucoryx*) [31], 16.8% for the giant panda (*Ailuropoda melanoleuca*) [32], 13.2% for North American bison (*Bison bison*) [33], 12.7% for the San Nicolas Island fox [21], while only 5.4% for the European mink (*Mustela lutreola*) [34]. Actually, considerable MHC variation was also evidenced at the *DQB* and MHC-I loci in the baiji. Six MHC-I, eight *DQB* alleles were separately examined in the same 17 baiji samples examined in this study, most of which were differed more than 20 nucleotides [35]–[36]. These combined evidences strongly suggest that the rapid population decline of baiji in the past decades have not resulted in significant decrease of MHC gene variability. In other words, abundant genetic diversity at adaptive MHC loci is still preserved in this endangered population.

**Table 5:** Genetic variation at the *DRB* exon 2 of some endangered or near threatened species.

Species	Status	Locations	sample size	Number of alleles	Observed heterozygosity	%N	%A	Reference
Baiji, *Lipotes vexillifer*	Critically Endangered	The Yangtze River, China	17	9 (215)	0.71	12.2 (1–57)	23.9 (0–30)	This study
Vaquita, *Phocoena sinus*	Critically Endangered	Upper Gulf of California	29	2 (210)	0.37	0.5(1)	1.5(1)	[21]
San Nicolas Island fox*Urocyon littoralis*	Critically Endangered	California Channel Island	152	3(267)	0.36	7.5 (13–24)	12.7 (9–14)	[21]–[22]
Giant panda*Ailuropoda melanoleuca*	Endangered	Sichuan and Shanxi, China	60	7 (282)	0.62	10.6(7–47)	16.8(4–25)	[36]
Beluga *Delphinapterus leucas*	Near Threatened	St. Lawrence Estuary, Bering Sea,Hudson Strait, Baffin Bay	313	8(238)	NA	7.2(1–25)	17.5(1–19)	[34]
North American bison *Bison bison*	Near Threatened	South Dakota and Montana	20	9(250)	NA	8.55(NA)	13.22(3–19)	[37]
European mink *Mustela lutreola*	Endangered	Estonian, Russia	20	9(229)	NA	6.2(4–25)	5.4(2–11)	[38]

The information on all studies derived from DRB sequence analysis includes sample size, number of alleles, length in base pairs (bp) of the DNA fragment analysed in parentheses, observed heterozygosity, average percentage nucleotide (%N) and amino acid (%A) divergence between alleles. In parentheses are the minimum and maximum number of nucleotide (or amino acid, respectively) differences observed between pairs of alleles.

### Balancing selection maintaining MHC allelic diversity

The unexpectedly high levels of MHC allelic diversity in the baiji may be partly attributable to possibility of severe pathogens and parasites. This prediction was evidenced in humans [37] and wild fish species [38]–[39]. These populations exposed to high parasite pressure, in terms of high parasite species richness, may maintain a high genetic diversity of the exon 2 of the MHC genes (presenting the PBR). Initial studies suggested that reduced pathogenic pressure in marine as compared with terrestrial environments might explain limited MHC variation in marine mammals [40]–[41]. However, recent assessments showed that cetacean MHC diversity was comparable to that in the terrestrial mammals [35]–[36], [42]–[44], arguing against reduced pathogenic pressure acting on marine mammal MHC genes. In the last 20 years, thousands of marine mammals have died due to epizootics caused by viral infections [45]. There was evidence of pathogen infections in the baiji that some helminths were found in the content of the stomach in the incidentally captured/killed baiji individuals. As the result, considerable *DRB* genetic variation was maintain in the baiji.

On the other hand, it has been suggested that selection may retain MHC diversity even when population bottlenecks lead to the loss of genetic variation elsewhere in the genome, exemplified by an endangered island fox which retained some MHC variation in spite of the virtual lack of neutral variation [46]. Similar pattern was found in the critically endangered baiji. In our previous study, the baiji had low level of genetic variability at neutral markers such as mitochondrial control region, with four haplotypes identified in 20 samples and only three variable sites in a total of 428 bp [47]. However, as stated above, there was considerable sequence variation at the adaptive MHC genes, such as *DRB*, *DQB*, and MHC-I loci. Moreover, several lines of evidences support that balancing selection plays a role in maintaining MHC sequence polymorphism at the *DRB*, MHC-I and *DQB* loci. First, the unexpectedly high level of *DRB* allele variation in the baiji was concentrated in PBR sites with an excess of nonsynonymous substitutions in PBR, which was considered as a primary indication of balancing selection. This inference was further evidenced by the result obtained from comparisons of various models of codon evolution (Table 3). Models allowing for positive selection offered a relatively better fit to the data than the single ω model. Second, a high level of divergence among MHC sequences within a population is suggested as another evidence that balancing selection is acting to maintain MHC variation [48]. For example, the *DRB* sequences were highly divergent with up to 26.5% (57/215) and 42.3% (30/71) at the nucleotide and amino acid levels, respectively. The number of pairwise nucleotide differences between alleles ranged from six (Live-I*04 vs. Live-I*06) to 30 (Live-I*05 vs. Live-I*06) and amino acid replacements ranged from 4 (Live-I*03 vs. Live-I*05) to 18 (Live-I*03 vs. Live-I*04). Third, balancing selection was also apparent in the pattern of trans-species polymorphism in phylogenetic reconstructions. Trans-species polymorphism is polymorphism predates speciation events, whereby allelic lineages are passed from species to species and persist over long periods of evolutionary time [49]. Thus, in the phylogenetic tree, alleles did not form a species-specific clade but intermixed with sequences from other species. This was evidenced in phylogenetic trees of the baiji at the *DRB*, *DQB* and MHC-I loci [35]–[36]. For example, some sequences of the baiji clustered with those of the finless porpoises at the *DRB* locus, e.g. Live-DRB*01, *03*, *05* clustering with Neph-DRB*06, *02*, *09*, respectively, etc.

Besides, two main types of balancing selection were suggested to maintain the unusually high level of MHC polymorphism: heterozygote advantage, rare-allele advantage. The heterozygote advantage hypothesis assumes that heterozygous individuals are favoured as they are able to present a broader array of antigens, and therefore resist a broader array of pathogens than homozygotes [50]. The rare-allele advantage hypothesis proposes that parasite antigenicity will be selected to exploit MHC-based immune response defect of the most common host genotype. This would decrease the relative fitness of the common host genotypes and provide a selective advantage to new, rare MHC alleles [51]–[52]. In the present study, the observed heterozygosity (0.71) was less than expected heterozygosity (0.83), which is disaccord with the heterozygote advantage hypothesis. In contrast, five *DRB* alleles, i.e. Live-DRB*05, *06*, *07*, *08*, *09*, were identified only in the two or three baiji samples with frequency ranging from 0.029 to 0.088. This pattern might in line with the mechanism of rare-allele advantage.

The maintenance of polymorphism within populations is dependent on the product of selection intensity [53]. It is estimated that the selection intensity at the human *DRB* locus (*s* = 0.019) is at least two times higher than for *DQB* (*s* = 0.0085) [54]. Munguia-Vega et al. [20] compared different MHC loci polymorphism in different species showed that the strength of selection acting at the *DQ* loci was generally insufficient to maintain variation in small populations isolated during hundred or thousand generations. However, on most cases some functional variation was still retained at the *DRB* locus, consistent with higher selection intensity at this locus. This agrees with theoretical models [53] that suggest moderate or strong balancing selection on small populations can oppose random drift and maintain polymorphism during thousands of generations. This theoretical model has been evidenced by Aguliar et al. [46]. Their empirical and simulation results suggested that intense balancing selection at the MHC may have allowed the persistence of variation in San Nicolas foxes despite strong genetic drift. For the baiji, an isolated population, still has unexpectedly high levels of *DRB* allelic diversity. Consequently, balancing selection at the MHC must have been intense in the baiji to maintain high levels of variation despite its population size rapidly declined in the past few decades.

Additionally, intragenic recombination was posed to provide another mechanism that regenerates MHC allelic diversity [24]. In this study, although recombination was detected in at least four of the 9 *Live-DRB* alleles, and two DNA segments (positions 83–198 and positions 146–189) may have acted as hot spots for recombination. However, the allele Live-DRB*01, *03*, *09* were came from one of the hot spots (positions 83–198), and the three alleles were substantially different, suggesting the mutation of alleles might have occurred after recombination. If so, the intragenic recombination must have been very ancient. Thus, intragenic recombination was likely not as major mechanism for the baiji *DRB* polymorphism.

The significant level of genetic variability at MHC loci, associated with apparent action of balancing selection on this gene, strongly suggested that the baiji, although had a very small population size and dramatically declined in the past decades, still had a strong adaptability and evolutionary potential. In other words, balancing selection might have overpowered genetic drift as the main driving force in shaping the genetic composition of the baiji. The dramatic declining trend or the current nearly extinct status of the baiji, therefore, seems to be dependent mainly on factors outside their genetic makeup. This finding further extends our understanding of the mechanism causing the endangerment or extinction of the baiji. Turvey et al. conducted an analysis of spatial and temporal extinction dynamics in the baiji and found that the population decline of baiji was not associated with any major contraction in geographical range across the middle–lower Yangtze drainage, even in the decade immediately before probable global extinction of this species [4]. In summary, we may conclude that the extinction risk in baiji was seemingly not related to evidences of genetic collapse.

## Materials and Methods

### Ethics Statement

All the baiji samples used in our study were collected from dead individuals in the wild so that no ehics statement is required. Voucher specimens are preserved at Jiangsu Key Laboratory for Biodiversity and Biotechnology, College of Life Sciences, Nanjing Normal University (NNU), China.

### Sample collection and DNA extraction

A total of 17 baiji samples, 3 muscle and 14 skeletal, were collected from stranded or incidentally captured/killed individuals in the Yangtze River. Genomic DNA was extracted by using the DNeasy tissue kit (QIAGEN) for muscle samples and Gene clean for ancient DNA kit (Q. Biogene) for skeletal samples, respectively, following the manufacturer's protocol.

### PCR amplication, cloning and sequencing

A 215-bp fragment (exclusive of primer sequences) from exon 2 of the *DRB* gene was amplified with the forward primer DRBex2f2b (5′-CRGTTTAAGAGCGAGTGTC-3′) and reverse primer DRB61a (5′-CCGCTGCACTGTGAAGCT-3′) [42]. The conditions for the polymerase chain reaction (PCR), DNA cloning was denoted in previous studies (e.g. [42]–[43]). In this study, twelve to fifteen clones were picked for each cloned PCR product and sequenced in the forward and/or reverse directions using the BigDye Terminator Cycle Sequencing Ready Reaction Kit (ABI) on an ABI 3730 automated genetic analyzer.

### Data analysis

A new allele was identified only when it met the criteria summarized by Kennedy et al. [25]. The criteria are, when using DNA cloning and sequencing, there have to be at least three identical clones, identified in either two separate PCRs from the same individual or from PCRs from at least two different individuals. In accordance with the proposed nomenclature for MHC in nonhuman species [26], we designated the exon 2 alleles *Live-DRB* for the baiji *Lipotes vexillifer* with serial numbers attached.

In order to test if positive selection has acted on the *DRB* evolution in the baiji, we used two approaches. First, nonsynonymous (*d*_N_) and synonymous (*d*_S_) substitution rates were calculated for the PBR and non-PBR amino acid positions following the method of Nei and Gojobori [55] using the Jukes-Cantor correction for multiple substitutions, and the significance levels were determined by a Z-test using the MEGA 4 software [56]. Second, evidence for positive selection was assessed using the CODEML subroutine contained in the PAML 4 program suite [57]. Positive selection is indicated by ω = *d*_N_/*d*_S_>1. Six models implemented in this study are: M0 (one-ratio), which assumes the same ω ratio for all codons; M1a (nearly neutral), which assumes two categories of sites conserved (ω = 0) and neutral (ω = 1); M2a (positive selection), which adds a third class of sites with ω as free parameter, thus allowing for sites with ω>1; M3 (discrete), which assumes three site classes with the proportions (*p*0, *p*1, *p*2) and ω ratios (ω0, ω1, ω2) estimated from the data; M7 (beta), which assumes ω varies among sites according to a beta distribution with parameters p and q; and M8 (beta and ω), which adds an additional site class to the beta model to account for sites under positive selection [58]. The likelihood scores from these models were compared using the Akaike information criterion (AIC) [59], and the model with the smallest relative AIC score was selected as the best approximation to these data [60]. Posterior probabilities for site classes have been calculated by Bayes empirical Bayes (BEB) method in models M2a and M8 [61]. If the posterior means of ω for some site classes are >1 (calculated as the average of ω over all site classes weighted by the posterior probabilities), those sites are likely to be under positive selection [61]. In addition, amino acid positions involved in the peptide binding were identified by comparing with the peptide binding groove structure of the human class II molecules [27].

Detection of potential recombinant sequences, identification of likely parental sequences, and localization of possible recombination break points were determined using the Recombination Detection Program (RDP) version 3 (http://darwin.uvigo.es/rdp/rdp.html) [62] using six different automated methods (RDP [63], GENECONV [64], MAXICHI [65], BOOTSCAN [66] SISCAN [67], CHIMAERA [68]). Multiple-comparison-corrected P-value cutoff of 0.05 was used throughout. Default parameters (highest acceptable probability value = 0.05) were used except that the general window size was set to 80 nt.

Phylogenetic analysis was carried out with confirmed alleles using nucleotide sequences of exon 2. The neighbor-joining (NJ) tree was reconstructed from the matrix of distances computed in PAUP 4.0b10 [69]. The robustness of the obtained tree topology was tested with 1,000 bootstrap replicates. Another tree was assembled using Bayesian analyses in MrBayes 3.1.2 [70]. Two independent runs of four Metropolis coupled Markov chain Monte Carlo (MCMC) simulations (three of them ‘heated’, temperature = 0.20) were each run for 1.0×10^6^ generations and sampled every 1,000 generations. The first 10% of trees were discarded as burn-in, resulting in 900 sampled trees. To calculate the posterior probability of each bipartition, the majority-rule consensus tree was computed from these 900 sampled trees. Sequences of the *Bison bison* (DQ354665) and *Hippopotamus amphibious* (EF017820) were used as outgroups. The best-fit model was selected using the AIC criterion and likelihood scores automatically generated and compared using MODELTEST [71].

## References

[1] Reeves RR, Smith BD, Crespo EA, Notarbartolo di Sciara G (2003). Dolphins, Whales and Porpoises: 2002–2010 Conservation action plan for the world's cetaceans..

[2] Yang G, Bruford MW, Wei F, Zhou K (2007). Conservation options for the baiji: time for realism?. Conserv Biol.

[3] Turvey ST, Pitman RL, Taylor BL, Barlow J, Akamatsu T (2007). First human-caused extinction of a cetacean species?. Biol Lett.

[4] Turvey ST, Barrett LA, Hart T, Collen B, Hao YJ (2010). Spatial and temporal extinction dynamics in a freshwater cetacean.. Proc R Soc B.

[5] Zhou K (1958). The finding of *Lipotes vexillifer* in the lower Yangtze River.. Chin Sci Bull.

[6] Zhou K, Qian W, Li Y (1977). Studies on the distribution of baiji, *Lipotes vexillifer* Miller.. Acta Zool Sinica.

[7] Zhou KY (1982). Conservation of the Baiji.. J Nanjing Normal Univ Nat Sci.

[8] Zhang X, Wang D, Liu R, Wei Z, Hua Y (2003). The Yangtze River dolphin or baiji (*Lipotes vexillifer*): population status and conservation issues in the Yangtze River, China.. Aquat Conserv Mar Freshw Ecosyst.

[9] Zhou K, Wang X (1994). Brief review of passive fishing gear and incidental catches of small cetaceans in Chinese waters.. Rep Int Whal Commn Special Issue.

[10] Zhou K, Sun J, Gao A, Wursig B (1998). Baiji (*Lipotes vexillifer*) in the lower Yangtze River: movements, numbers threats and conservation needs.. Aquat Mamm.

[11] IUCN 2011 (2009). IUCN Red List of Threatened Species.

[12] Vassilakos D, Natoli AA, Dahlheim M, Hoelzel AR (2009). Balancing and directional selection at exon-2 of the MHC DQB1 locus among populations of Odontocete cetaceans.. Mol Biol Evol.

[13] Van Bressem MF, Van Waerebeek K, Raga AJ (1999). A review of virus infections of cetaceans and the potential impact of morbilliviruses, poxviruses and pappilomaviruses on host population dynamics.. Dis Aquat Org.

[14] Klein J (1986). Natural History of the Major Histocompatibility Complex.

[15] Bernatchez L, Landry C (2003). MHC studies in nonmodel vertebrates: what have we learned about natural selection in 15 years?. J Evol Biol.

[16] Piertney SB, Oliver Mk (2006). The evolutionary ecology of the major histocompatibility complex.. Heredity.

[17] O'Brien SJ, Evermann JF (1988). Interactive influence of infectious disease and genetic diversity in natural populations.. Trends Ecol Evol.

[18] Lochmiller RL (1996). Immunocompetence and animal population regulation.. Oikos.

[19] Amills M, Jime'nez N, Jordana J, Riccardi A, Ferna'ndez-Arias A (2004). Low diversity in the major histocompatibility complex class II DRB1 gene of the Spanish ibex, *Capra pyrenaica*.. Heredity.

[20] Munguia-Vega A, Esquer-Garrigos Y, Rojas-Bracho L, Vazquez-Juarez R, Castro-Prieto A (2007). Genetic drift vs. natural selection in a long-term small isolated population: major histocompatibility complex class II variation in the Gulf of California endemic porpoise (*Phocoena sinus*).. Mol Ecol.

[21] Aguilar A, Roemer G, Debenham S, Binns M, Garcelon D (2004). High MHC diversity maintained by balancing selection in an otherwise genetically monomorphic mammal.. Proc Natl Acad Sci USA.

[22] Yuhki N, O'Brien SJ (1990). DNA variation of the mammalian major histocompatibility complex reflects genomic diversity and population history.. Proc Natl Acad Sci USA.

[23] Edwards SV, Hedrick PW (1998). Evolution and ecology of MHC molecules: from genomics to sexual selection.. Trends Ecol Evol.

[24] Richman AD, Herrera LG, Nash D, Schierup HM (2003). Relative roles of mutation and recombination in generating allelic polymorphism at an MHC class II locus in *Peromyscus maniculatus*.. Genet Res Camb.

[25] Kennedy LJ, Ryvar R, Gaskell RM, Addie DD, Willoughby K (2002). Sequence analysis of MHC DRB alleles in domestic cats from the United Kingdom.. Immunogenetics.

[26] Klein J, Bontrop RE, Dawkins RL, Erlich HA, Gyllensten UB (1990). Nomenclature for the major histocompatibility complexes of different species: a proposal.. Immunogenetics.

[27] Brown JH, Jardetzky TS, Gorga JC, Stern LJ, Urban RG (1993). 3-Dimensional structure of the human class-II histocompatibility antigen HLADR1.. Nature.

[28] Zhou KY, Perrin WF, Würsig B, Thewissen JGM (2002). Baiji.. Encyclopedia of Marine Mammals.

[29] Weber DS, Stewart BS, Schienman J, Lehman N (2004). Major histocompatibility complex variation at three class II loci in the northern elephant seal.. Mol Ecol.

[30] Murray BW, White BN (1998). Sequence variation at the major histocompartibility complex DRB loci in beluga (*Delphinapterus leucas*) and narwhal (*Monodon monoceros*).. Immunogenetics.

[31] Hedrick PW, Parker KM, Gutierrez Espeleta GA, Rattink A, Lievers K (2000). Major histocompatibility complex variation in the *Arabian oryx*.. Evolution.

[32] Wan QH, Zhu L, Wu H, Fang SG (2006). Major histocompatibility complex class II variation in the giant panda (*Ailuropoda melanoleuca*).. Mol Ecol.

[33] Mikko S, Spencer M, Morris B, Stabile S, Basu T (1997). A comparative analysis of the Mhc *DRB3* polymorphism in the American Bison (*Bison bison*).. J Hered.

[34] Becker L, Nieberg C, Jahreis K, Peters E (2009). MHC class II variation in the endangered European mink *Mustela lutreola* (L. 1761)-consequences for species conservation.. Immunogenetics.

[35] Xu SX, Chen BY, Zhou KY, Yang G (2008). High sequence similarity at three MHC loci between baiji and finless porpoise: trans-species or convergent evolution?. Mol Phylogenet Evol.

[36] Yang G, Yan J, Zhou KY, Wei FW (2005). Sequence variation and gene duplication at MHC DQB loci of Baiji (*Lipotes vexillifer*), a Chinese river dolphin.. J Hered.

[37] Prugnolle F, Manica A, Charpentier M, Gue'gan J-F, Guernier V (2005). Pathogen-driven selection and worldwide HLA class I diversity.. Curr Biol.

[38] Wegner KM, Reusch TB, Kalbe M (2003). Multiple parasites are driving major histocompatibility complex polymorphism in the wild.. J Evol Biol.

[39] Simkova A, Ottova E, Morand S (2006). MHC variability, lifetraits and parasite diversity of European cyprinid fish.. Evol Ecol.

[40] Trowsdale J, Gorves V, Arnason A (1989). Limited MHC polymorphism in whales.. Immunogenetics.

[41] Slade R (1992). Limited MHC polymorphism in the southern elephant seal: implications for MHC evolution and marine mammal population biology.. Proc R Soc Lond B.

[42] Baker CS, Vant MD, Dalebout ML, Lento GM, O'Brien SJ (2006). Diversity and duplication of *DQB* and *DRB*-like genes of the MHC in baleen whales (suborder: Mysticeti).. Immunogenetics.

[43] Xu S, Ren W, Zhou X, Zhou K, Yang G (2010). Sequence polymorphism and geographical variation at a positively selected MHC-DRB gene in the finless porpoise (*Neophocaena phocaenoides*): implication for recent differentiation of the Yangtze finless porpoise?. J Mol Evol.

[44] Xu SX, Sun P, Zhou KY, Yang G (2007). Sequence variability at three MHC loci of finless porpoises (*Neophocaena phocaenoides*).. Immunogenetics.

[45] Van Bressem MF, Van Waerebeek K, Raga AJ (1999). A review of virus infections of cetaceans and the potential impact of morbilliviruses, poxviruses and pappilomaviruses on host population dynamics.. Dis Aquat Org.

[46] Aguilar A, Roemer G, Debenham S, Binns M, Garcelon D, Wayne RK (2004). High MHC diversity maintained by balancing selection in an otherwise genetically monomorphic mammal.. Proc Natl Acad Sci USA.

[47] Yang G, Liu S, Ren WH, Zhou K, Wei F (2003). Mitochondrial control region variability of baiji and the Yangtze finless porpoises, two sympatric small cetaceans in the Yangtze river.. Acta Theriol.

[48] Richardson DS, Westerdahl H (2003). MHC diversity in two Acrocephalus species: the outbred great reed warbler and the inbred Seychelles warbler.. Mol Ecol.

[49] Klein J (1987). Origin of major histocompatibility complex polymorphism-the transspecies hypothesis.. Hum Immunol.

[50] Hughes AL, Nei M (1989). Nucleotide substitution at major histocompatibility complex class II loci: evidence for overdominant selection.. Proc Natl Acad Sci USA.

[51] Clarke BC, Kirby DRS (1966). Maintenance of histocompatibility polymorphism.. Nature.

[52] Takahata N, Nei M (1990). Allelic genealogy under overdominant and frequency-dependent selection and polymorphism of major histocompatibility complex loci.. Genetics.

[53] Nevo E, Kirzher V, Beiles A, Karol A (1997). Selection versus random drift: long-term polymorphism persistence in small populations (evidence and modelling).. Phil Trans Royal Soc B.

[54] Satta Y, O'hUigin C, Takahata N, Klein J (1994). Intensity of natural selection at the major histocompatibility complex loci.. Proc Natl Acad Sci.

[55] Nei M, Gojobori T (1986). Simple methods for estimating the numbers of synonymous and nonsynonymous nucleotide substitutions.. Mol Biol Evol.

[56] Tamura K, Dudley J, Nei M, Kumar S (2007). MEGA 4: Molecular Evolutionary Genetics Analysis (MEGA) software version 4.0.. Mol Biol Evol.

[57] Yang ZH (2007). PAML 4: Phylogenetic Analysis by Maximum Likelihood.. Mol Biol Evol.

[58] Yand Z, Bielawsji JP (2000). Statistical methods for detecting molecular adaptation.. Tree.

[59] Akaike H (1974). A new look at the statistical model indentification.. IEEE Trans Automat Control.

[60] Burnham KP, White GC (2002). Evaluation of some random effects methodology applicable to bird ringing data.. J Appl Stat.

[61] Yang ZH, Wong WSW, Nielsen R (2005). Bayes empirical Bayes inference of amino acid sites under positive selection.. Mol Biol Evol.

[62] Martin DP, Williamson C, Posada D (2005). RDP2: recombination detection and analysis from sequence alignments.. Bioinformatics.

[63] Martin D, Rybicki E (2000). RDP: detection of recombination amongst aligned sequences.. Bioinformatics.

[64] Padidam M, Sawyer S, Fauquet CM (1999). Possible emergence of new geminiviruses by frequent recombination.. Virology.

[65] The MaxChi method: Maynard Smith J (1992). Analyzing the mosaic structure of genes.. J Mol Evol.

[66] Martin DP, Posada D, Crandall KA, Williamson C (2005). A modified bootscan algorithm for automated identification of recombinant sequences and recombination breakpoints.. AIDS Res Hum Retroviruses.

[67] Gibbs MJ, Armstrong JS, Gibbs AJ (2000). Sister-Scanning: a Monte Carlo procedure for assessing signals in recombinant sequences.. Bioinformatics.

[68] Posada D, Crandall KA (2001). Evaluation of methods for detecting recombination from DNA sequences: Computer simulations.. Proc Natl Acad Sci.

[69] Swofford DL (2003). PAUP*: Phylogenetic Analysis Using Parsimony (*and other methods). Version 4.0b10.

[70] Ronquist F, Huelsenbeck JP (2003). MRBAYES 3: Bayesian phylogeneticinference under mixed models.. Bioinformatics.

[71] Posada D, Crandall KA (1998). Modeltest: Testing the model of DNA substitution.. Bioinformatics.

